# Supplementary material for: An extreme mutational hotspot in nlpD depends on transcriptional induction of rpoS
Source: PLoS Genet. 2025 Jan 31;21(1):e1011572. doi: 10.1371/journal.pgen.1011572 (PMC11838912; doi:10.1371/journal.pgen.1011572)
Supplement: S1 Fig — The TSS of rpoS is approximately 12 bp downstream of the position of the C565T mutation (Red C). Circles represent the TSS from individually cloned reverse-transcription fragments from RNA expressed in stationary phase cultures of either SBW25 or SBW25 Δwss (see S1 Table for all sequenced TSS positions). Cloned fragments were derived from one of three replicate cultures (indicated by either white, grey or black circles). The position of the mapped TSS of rpoS in P. aeruginosa is marked blue [22]. Triangles represent the consensus ‘gearbox’ promoter motif [60]. (PDF) [file pgen.1011572.s001.pdf]

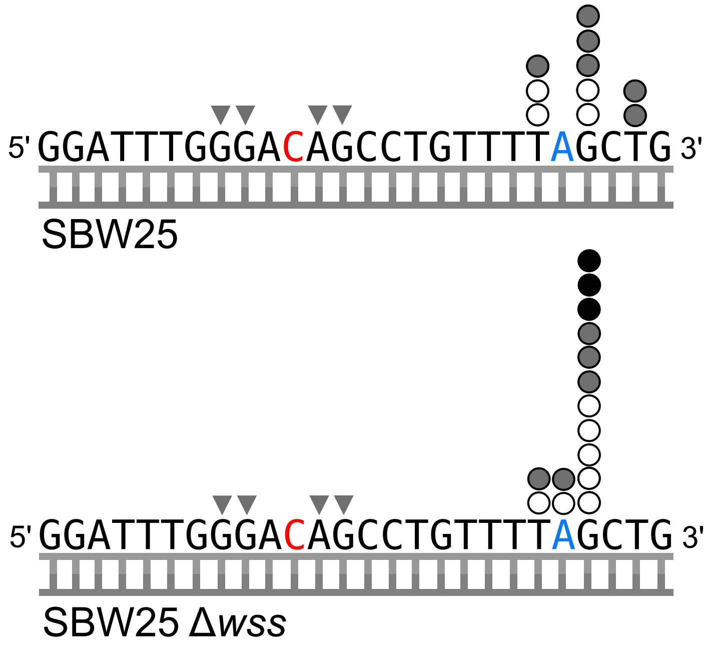

S1 Fig. The position of the C565T mutation relative to the transcriptional start site (TSS). The TSS of *rpoS* is approximately 12 bp downstream of the position of the C565T mutation (Red C). Circles represent the TSS from individually cloned reverse-transcription fragments from RNA expressed in stationary phase cultures of either SBW25 or SBW25 Δ*wss* (see S1 Table for all sequenced TSS positions). Cloned fragments were derived from one of three replicate cultures (indicated by either white, grey or black circles). The position of the mapped TSS of *rpoS* in *P. aeruginosa* is marked blue [1]. Triangles represent the consensus 'gearbox' promoter motif [2].

References

1. Fujita M, Tanaka K, Takahashi H, Amemura A. Transcription of the principal sigma-factor genes, *rpoD* and *rpoS*, in *Pseudomonas aeruginosa* is controlled according to the growth phase. Mol Microbiol. 1994;13(6):1071-7. doi: 10.1111/j.1365-2958.1994.tb00498.x. PubMed PMID: 7531806.

2. Vicente M, Kushner SR, Garrido T, Aldea M. The role of the 'gearbox' in the transcription of essential genes. Mol Microbiol. 1991;5(9):2085-91. doi: 10.1111/j.1365-2958.1991.tb02137.x. PubMed PMID: 1766382.
