# Supplementary material for: An extreme mutational hotspot in nlpD depends on transcriptional induction of rpoS
Source: PLoS Genet. 2025 Jan 31;21(1):e1011572. doi: 10.1371/journal.pgen.1011572 (PMC11838912; doi:10.1371/journal.pgen.1011572)
Supplement: S2 Fig — The rpoSp-kan reporter construct was made to express Green or mScarlet fluorescent proteins, and evolved in genotypes SBW25 or SBW25 ΔpsrA to feature a C565T mutation in the rpoSp-kan reporter. Presented is the relative fitness of genotypes with C565T rpoSp-kan vs unmutated rpoSp-kan. Competitions were initiated with a 1:1 ratio and grown in a manner similar to fluctuation assays. Reciprocal pairwise competitions were used, with the C565T mutation in either a GFP (green) or mScarlet (red) background. A significant lower fitness was measured for the C565T mutant in rpoSp-kan in both backgrounds (Wilcoxon signed rank exact test of either SBW25 or SBW25 ΔpsrA, V = 29, p-value = 0.04431). Each competition involved 16 replicates, black dots represent the mean values and error bars one standard deviation from the mean. (PDF) [file pgen.1011572.s002.pdf]

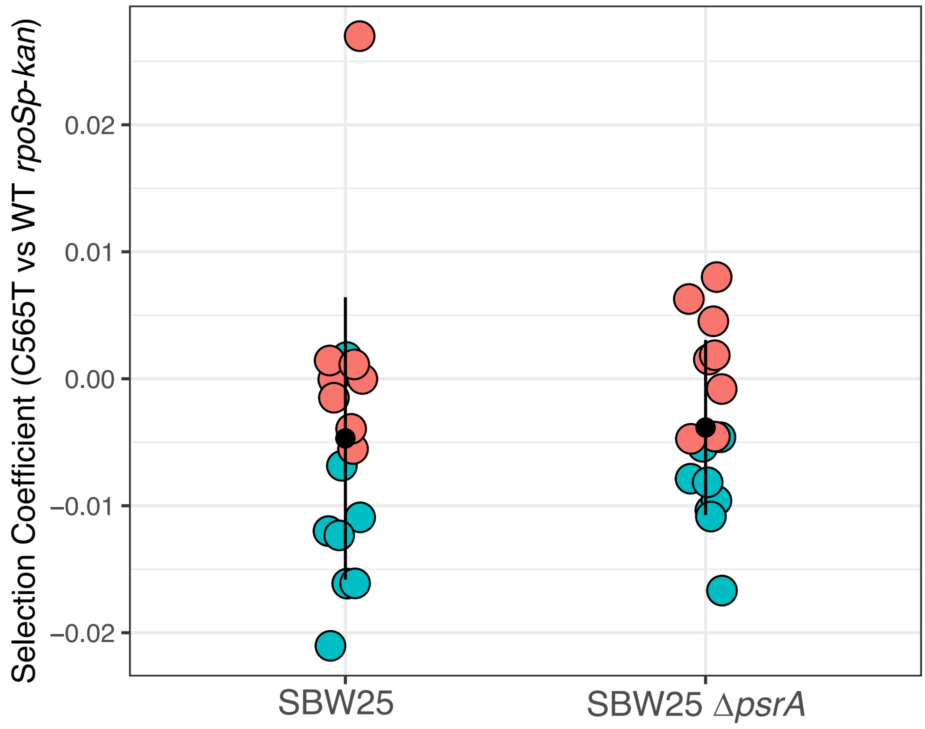

S2 Fig: Relative fitness of SBW25 genotypes with C565T mutated *rpoSp-kan* reporters vs non-mutant reporter genotypes. The *rpoSp-kan* reporter construct was made to express Green or mScarlet fluorescent proteins, and evolved in genotypes SBW25 or SBW25 *ΔpsrA* to feature a C565T mutation in the *rpoSp-kan* reporter. Presented is the relative fitness of genotypes with C565T *rpoSp-kan* vs unmutated *rpoSp-kan*. Competitions were initiated with a 1:1 ratio and grown in a manner similar to fluctuation assays. Reciprocal pairwise competitions were used, with the C565T mutation in either a GFP (green) or mScarlet (red) background. A significant lower fitness was measured for the C565T mutant in *rpoSp-kan* in both backgrounds (Wilcoxon signed rank exact test of either SBW25 or SBW25 *ΔpsrA*, V = 29, p-value = 0.04431). Each competition involved 16 replicates, black dots represent the mean values and error bars one standard deviation from the mean.
