# Supplementary material for: An extreme mutational hotspot in nlpD depends on transcriptional induction of rpoS
Source: PLoS Genet. 2025 Jan 31;21(1):e1011572. doi: 10.1371/journal.pgen.1011572 (PMC11838912; doi:10.1371/journal.pgen.1011572)
Supplement: S3 Fig — SBW25 was grown from a small inoculum (~1000 CFU mL-1) for 14, 18 and 22 hrs and samples were used to measure CFU concentrations and processed (as per Fig 3) to measure induction of transcription from rpoSp relative to a region in nlpD upstream of rpoSp. Similar levels of activity from rpoSp were measured after 18 and 22 hrs of growth, and fluctuation assays used cultures grown for 22 hrs to ensure cells were in stationary phase. (PDF) [file pgen.1011572.s003.pdf]

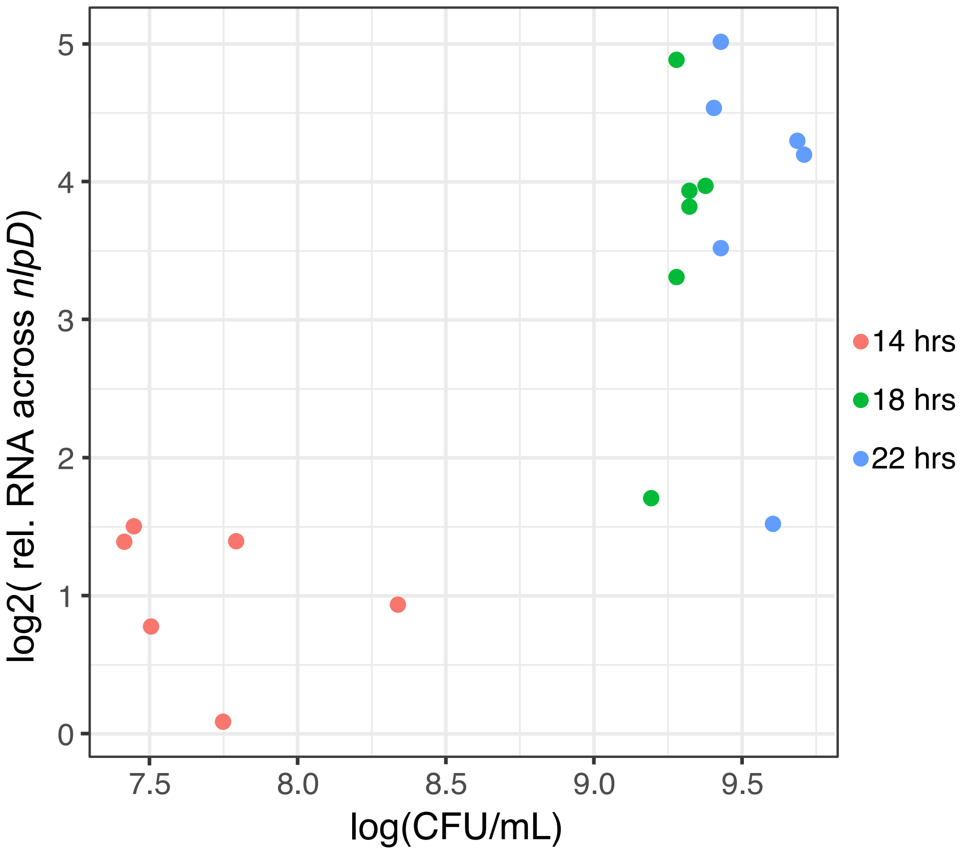

S3 Fig: Growth of SBW25 causes induction of transcription from *rpoSp*. SBW25 was grown from a small inoculum (~1000 CFU mL^{-1}) for 14, 18 and 22 hrs and samples were used to measure CFU concentrations and processed (as per Fig 3) to measure induction of transcription from *rpoSp* relative to a region in *nlpD* upstream of *rpoSp*. Similar levels of activity from *rpoSp* were measured after 18 and 22 hrs of growth, and fluctuation assays used cultures grown for 22 hrs to ensure cells were in stationary phase.
