# Supplementary material for: An extreme mutational hotspot in nlpD depends on transcriptional induction of rpoS
Source: PLoS Genet. 2025 Jan 31;21(1):e1011572. doi: 10.1371/journal.pgen.1011572 (PMC11838912; doi:10.1371/journal.pgen.1011572)
Supplement: S4 Fig — Fluctuation assays were performed as per Fig 3, except sampling occurred at earlier time points (four replicates per grouped time point). Samples at earlier time points ~15.8 hrs and ~17.3 hrs had significantly lower frequencies of the C565T mutation in the rpoSp-kan construct compared to the final time point of ~21.8 hrs (p = 0.04 and p = 0.03 respectively; one-way ANOVA, F(5,8.301) = 3.874, p = 0.04, pairwise difference assessed with Tukey HSD). (PDF) [file pgen.1011572.s004.pdf]

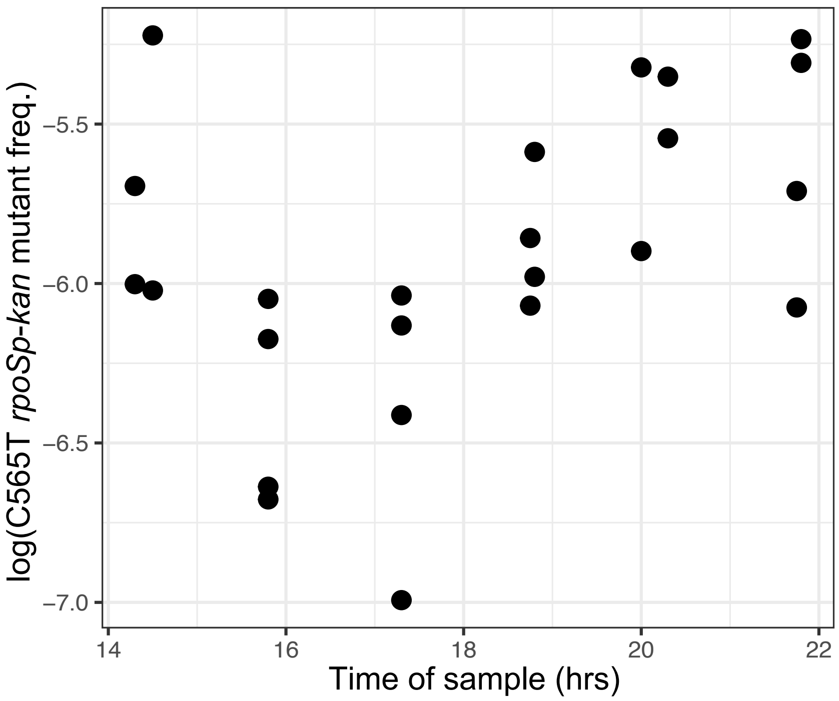

S4 Fig: The frequency of C565T mutant reporter strains as cultures approach stationary phase. Fluctuation assays were performed as per Fig 3, except sampling occurred at earlier time points (four replicates per grouped time point). Samples at earlier time points ~15.8 hrs and ~17.3 hrs had significantly lower frequencies of the C565T mutation in the *rpoSp-kan* construct compared to the final time point of ~21.8 hrs ($p=0.04$ and $p=0.03$ respectively; one-way ANOVA, $F(5,8.301) = 3.874$, $p = 0.04$, pairwise difference assessed with Tukey HSD).
