# Supplementary material for: An extreme mutational hotspot in nlpD depends on transcriptional induction of rpoS
Source: PLoS Genet. 2025 Jan 31;21(1):e1011572. doi: 10.1371/journal.pgen.1011572 (PMC11838912; doi:10.1371/journal.pgen.1011572)
Supplement: S5 Fig — SBW25 Δwss (nlpD anc) and SBW25 Δwss nlpDQ189W (which has a TGG sequence for nlpD codon 189 and a likely alteration of the rpoSp sequence) were grown to stationary phase, and mRNA was extracted, reverse transcribed and the regions up and down stream of rpoSp were used as templates for qPCR. SBW25 Δwss has levels consistent with other measures of induced transcription from rpoSp, while the C565T A566G mutation to rpoSp removes any indication of transcription from rpoSp (i.e. there is no difference in the levels of transcript downstream compared to upstream of rpoSp). Black dots represent the mean values of five biological replicates (gray dots) and error bars one standard deviation from the mean. (PDF) [file pgen.1011572.s005.pdf]

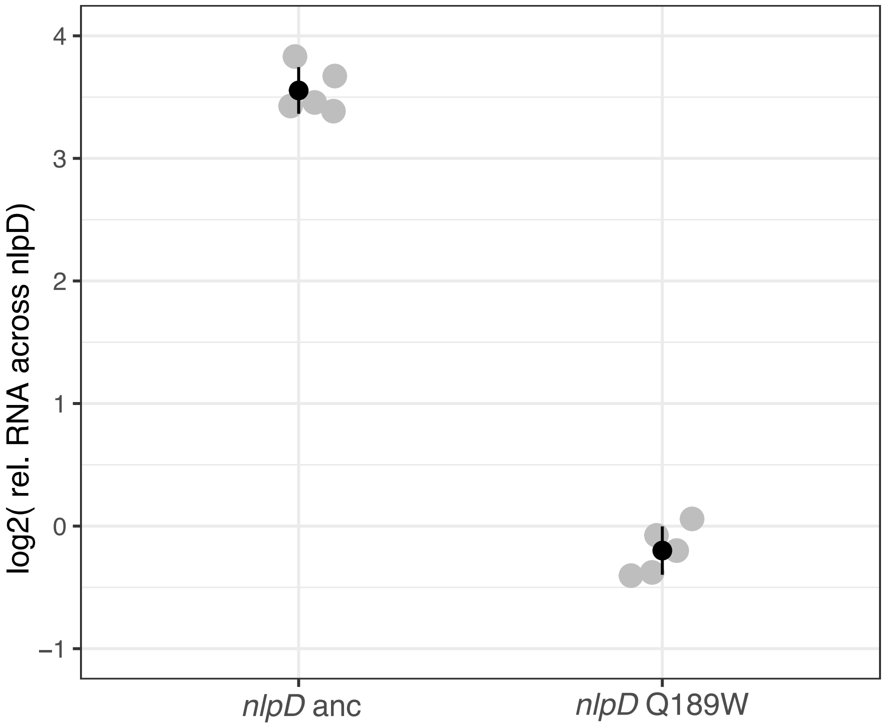

S5 Fig: The Q189W mutation prevents transcription from *rpoSp* for cells in stationary phase. SBW25 Δ*wss* (*nlpD* anc) and SBW25 Δ*wss nlpD*Q189W (which has a TGG sequence for *nlpD* codon 189 and a likely alteration of the *rpoSp* sequence) were grown to stationary phase, and mRNA was extracted, reverse transcribed and the regions up and down stream of *rpoSp* were used as templates for qPCR. SBW25 Δ*wss* has levels consistent with other measures of induced transcription from *rpoSp*, while the C565T A566G mutation to *rpoSp* removes any indication of transcription from *rpoSp* (i.e. there is no difference in the levels of transcript downstream compared to upstream of *rpoSp*). Black dots represent the mean values of five biological replicates (gray dots) and error bars one standard deviation from the mean.
