# Supplementary material for: An extreme mutational hotspot in nlpD depends on transcriptional induction of rpoS
Source: PLoS Genet. 2025 Jan 31;21(1):e1011572. doi: 10.1371/journal.pgen.1011572 (PMC11838912; doi:10.1371/journal.pgen.1011572)
Supplement: S1 Table — (DOCX) [file pgen.1011572.s006.docx]

**S1 Table. Mutational positions and residue changes of nlpD mutants arising from populations of SBW25 ∆wss nlpD (Q189W) grown in statically incubated microcosms.** Underlined isolates were reconstructed for fitness assays. Numbers within brackets represents amino acids until a stop codon is reached. Data derives from raw data for Fig 1.

| **Isolate** | **Nucleotide change** | **Amino acid change** |
| --- | --- | --- |
| 1 | G553A | G185R |
| 2 | Δ487-570 | Δ163-190 (Δ 28aa) |
| 3 | C603G | Y201* |
| 4, 14, 27 | A680G | H227R |
| 5, 6, 7, 9, 13, 16, 20, 22 | G566A | Q189* |
| 8 | Δ623-634 | Δ208-212 (Δ ATAS) |
| 10 | C775G | H259N |
| 11 | G483A | W161* |
| 12 | Δ620 | ΔR207(146) |
| 15 | Δ462 | P154(13) |
| 17 | G677A | G226D |
| 18 | C212T | A71V |
| 19 | C787T | R263C |
| 21, 26, 31 | A602G | Y201C |
| 23 | C811T | P271S |
| 24 | C817T | Q273* |
| 25, 30 | G539A | G180D |
| 28 | - | - |
| 29 | G493A | G165R |
| 32 | T91C | C31R |
| 33 | Δ396-397 | P132(43) |
| 34 | Δ141-144 | A47(6) |
| 35 | C484T | P162S |
| 36 | G595T | V199L |
| 37 | C49T | R17* |
| 38 | A809G | D270G |
