# Supplementary material for: An extreme mutational hotspot in nlpD depends on transcriptional induction of rpoS
Source: PLoS Genet. 2025 Jan 31;21(1):e1011572. doi: 10.1371/journal.pgen.1011572 (PMC11838912; doi:10.1371/journal.pgen.1011572)
Supplement: S2 Table — (DOCX) [file pgen.1011572.s007.docx]

**S2 Table: Positioning of the transcriptional start site (TSS) from total RNA extracts of SBW25 and SBW25 ∆*wss* using 5-prime RACE.** Data derived from raw data for S1 Fig.

| Genotype | MPB number of isolate | Replicate | Colony | Position in *nlpD* ORF |
| --- | --- | --- | --- | --- |
| SBW25 | MPB29692 | 1 | 1 | 666 |
| SBW25 | MPB29693 | 1 | 2 | 577 |
| SBW25 | MPB29694 | 1 | 3 | 591 |
| SBW25 | MPB29695 | 1 | 4 | 575 |
| SBW25 | MPB29696 | 1 | 5 | 644 |
| SBW25 | MPB29697 | 1 | 6 | 744 |
| SBW25 | MPB29698 | 1 | 7 | 575 |
| SBW25 | MPB29699 | 1 | 8 | 659 |
| SBW25 | MPB29700 | 1 | 9 | 648 |
| SBW25 | MPB29701 | 1 | 10 | 577 |
| SBW25 | MPB30639 | 2 | 1 | 577 |
| SBW25 | MPB30640 | 2 | 2 | 577 |
| SBW25 | MPB30641 | 2 | 3 | 575 |
| SBW25 | MPB30642 | 2 | 4 | 708 |
| SBW25 | MPB30643 | 2 | 5 | 659 |
| SBW25 | MPB30644 | 2 | 6 | 664 |
| SBW25 | MPB30645 | 2 | 7 | 579 |
| SBW25 | MPB30646 | 2 | 8 | 577 |
| SBW25 | MPB30647 | 2 | 9 | 698 |
| SBW25 | MPB30648 | 2 | 10 | 579 |
| SBW25 | MPB30649 | 3 | 1 | 696 |
| SBW25 | MPB30650 | 3 | 2 | 734 |
| SBW25 | MPB30651 | 3 | 3 | No product |
| SBW25 | MPB30652 | 3 | 4 | 729 |
| SBW25 | MPB30653 | 3 | 5 | 500 |
| SBW25 | MPB30654 | 3 | 6 | 697 |
| SBW25 | MPB30655 | 3 | 7 | 714 |
| SBW25 | MPB30656 | 3 | 8 | 698 |
| SBW25 | MPB30657 | 3 | 9 | 422 |
| SBW25 | MPB30658 | 3 | 10 | 698 |
| SBW25 ∆*wss* | MPB29704 | 1 | 1 | 577 |
| SBW25 ∆*wss* | MPB29705 | 1 | 2 | 491 |
| SBW25 ∆*wss* | MPB29706 | 1 | 3 | 577 |
| SBW25 ∆*wss* | MPB29707 | 1 | 4 | 577 |
| SBW25 ∆*wss* | MPB29708 | 1 | 5 | 577 |
| SBW25 ∆*wss* | MPB29709 | 1 | 6 | 577 |
| SBW25 ∆*wss* | MPB29710 | 1 | 7 | 756 |
| SBW25 ∆*wss* | MPB29711 | 1 | 8 | 576 |
| SBW25 ∆*wss* | MPB29712 | 1 | 9 | 377 |
| SBW25 ∆*wss* | MPB29713 | 1 | 10 | 575 |
| SBW25 ∆*wss* | MPB30659 | 2 | 1 | 577 |
| SBW25 ∆*wss* | MPB30660 | 2 | 2 | 575 |
| SBW25 ∆*wss* | MPB30661 | 2 | 3 | 577 |
| SBW25 ∆*wss* | MPB30662 | 2 | 4 | 698 |
| SBW25 ∆*wss* | MPB30663 | 2 | 5 | 577 |
| SBW25 ∆*wss* | MPB30664 | 2 | 6 | 663 |
| SBW25 ∆*wss* | MPB30665 | 2 | 7 | 648 |
| SBW25 ∆*wss* | MPB30666 | 2 | 8 | 577 |
| SBW25 ∆*wss* | MPB30667 | 2 | 9 | 576 |
| SBW25 ∆*wss* | MPB30668 | 2 | 10 | 698 |
| SBW25 ∆*wss* | MPB30669 | 3 | 1 | 711 |
| SBW25 ∆*wss* | MPB30670 | 3 | 2 | 643 |
| SBW25 ∆*wss* | MPB30671 | 3 | 3 | 698 |
| SBW25 ∆*wss* | MPB30672 | 3 | 4 | 577 |
| SBW25 ∆*wss* | MPB30673 | 3 | 5 | No product |
| SBW25 ∆*wss* | MPB30674 | 3 | 6 | 725 |
| SBW25 ∆*wss* | MPB30675 | 3 | 7 | 663 |
| SBW25 ∆*wss* | MPB30676 | 3 | 8 | 349 |
| SBW25 ∆*wss* | MPB30677 | 3 | 9 | 577 |
| SBW25 ∆*wss* | MPB30678 | 3 | 10 | 738 |
