# Supplementary material for: An extreme mutational hotspot in nlpD depends on transcriptional induction of rpoS
Source: PLoS Genet. 2025 Jan 31;21(1):e1011572. doi: 10.1371/journal.pgen.1011572 (PMC11838912; doi:10.1371/journal.pgen.1011572)
Supplement: S3 Table — (DOCX) [file pgen.1011572.s008.docx]

**S3 Table: Data used in calculating the C565T mutation rate using SBW25 *attTn7*::*rpoSp*-*kan* (genotype ‘WT *r-k’* below) and SBW25 ∆*psrA* *attTn7*::*rpoSp-kan* (genotype ‘∆*psrA* r-k’).** Data derives from raw data for Fig 4A.

| Genotype | Replicate | Trial | Final  CFU / mL (22 hrs) | CFU >3mm kanR colonies /mL | Fraction C565T  colonies | Fraction A564G  colonies | Frequency C565T colonies |
| --- | --- | --- | --- | --- | --- | --- | --- |
| WT *r-k* | 1 | 1 | 4.34E+09 | 2.89E+04 | 7 / 8 | 1/8 | 5.82E-06 |
| WT *r-k* | 2 | 1 | 2.80E+09 | 9.00E+03 | 6 / 8 | 1/8 | 2.41E-06 |
| WT *r-k* | 3 | 1 | 3.40E+09 | 1.10E+04 | 8 / 8 | 0/8 | 3.24E-06 |
| WT *r-k* | 4 | 1 | 3.86E+09 | 2.00E+04 | 5 / 8 | 0/8 | 3.24E-06 |
| WT *r-k* | 5 | 1 | 3.46E+09 | 1.56E+04 | 2 / 8 | 1/8 | 1.12E-06 |
| WT *r-k* | 6 | 1 | 2.50E+09 | 8.00E+03 | 6 / 8 | 2/8 | 2.40E-06 |
| WT *r-k* | 7 | 2 | 3.64E+09 | 3.33E+04 | 4 / 8 | 0/8 | 4.58E-06 |
| WT *r-k* | 8 | 2 | 3.28E+09 | 4.89E+04 | 4 / 8 | 0/8 | 7.45E-06 |
| WT *r-k* | 9 | 2 | 3.86E+09 | 4.22E+04 | 6 / 8 | 0/8 | 8.20E-06 |
| WT *r-k* | 10 | 2 | 3.86E+09 | 4.00E+04 | 3 / 8 | 0/8 | 3.89E-06 |
| WT *r-k* | 11 | 2 | 3.32E+09 | 1.18E+04 | 3 / 8 | 1/8 | 1.33E-06 |
| WT *r-k* | 12 | 2 | 3.34E+09 | 4.67E+04 | 6 / 8 | 0/8 | 1.05E-05 |
| ∆*psrA r-k* | 1 | 1 | 3.16E+09 | 8.22E+01 | 8 / 8 | 0/8 | 2.60E-08 |
| ∆*psrA r-k* | 2 | 1 | 3.78E+09 | 1.89E+02 | 8 / 8 | 0/8 | 5.00E-08 |
| ∆*psrA r-k* | 3 | 1 | 2.46E+09 | 6.44E+01 | 8 / 8 | 0/8 | 2.62E-08 |
| ∆*psrA r-k* | 4 | 1 | 3.08E+09 | 4.67E+01 | 7 / 7 | 0/7 | 1.52E-08 |
| ∆*psrA r-k* | 5 | 1 | 3.66E+09 | 1.00E+02 | 8 / 8 | 0/8 | 2.73E-08 |
| ∆*psrA r-k* | 6 | 1 | 2.28E+09 | 4.44E+01 | 8 / 8 | 0/8 | 1.95E-08 |
| ∆*psrA r-k* | 7 | 2 | 3.38E+09 | 4.51E+02 | 8 / 8 | 0/8 | 1.33E-07 |
| ∆*psrA r-k* | 8 | 2 | 3.68E+09 | 6.67E+01 | 8 / 8 | 0/8 | 1.81E-08 |
| ∆*psrA r-k* | 9 | 2 | 2.28E+09 | 7.11E+01 | 8 / 8 | 0/8 | 3.12E-08 |
| ∆*psrA r-k* | 10 | 2 | 3.28E+09 | 8.44E+01 | 8 / 8 | 0/8 | 2.57E-08 |
| ∆*psrA r-k* | 11 | 2 | 2.72E+09 | 6.22E+01 | 8 / 8 | 0/8 | 2.29E-08 |
| ∆*psrA r-k* | 12 | 2 | 2.94E+09 | 1.76E+02 | 8 / 8 | 0/8 | 5.97E-08 |
