# Supplementary material for: An extreme mutational hotspot in nlpD depends on transcriptional induction of rpoS
Source: PLoS Genet. 2025 Jan 31;21(1):e1011572. doi: 10.1371/journal.pgen.1011572 (PMC11838912; doi:10.1371/journal.pgen.1011572)
Supplement: Attachment — Submitted filename: 09_12_24_Response to reviewers comments.docx [file pgen.1011572.s010.docx]

**9_12_24**

**Manuscript ID: PGENETICS-D-24-01022R1**

**Authors response to reviewers for** “**An extreme mutational hotspot in *nlpD* depends on transcriptional induction of *rpoS*”. Authors responses are between underscores are in arial 11 font.**

The authors would like to thank all reviewers for their constructive and specific feedback. The reviewers appeared well engaged with the manuscript and left fair and constructive comments and have left us pleased with our choice of journal.

_

Reviewer's Responses to Questions

**Comments to the Authors:
Please note here if the review is uploaded as an attachment.**

**Reviewer #1**: I would firstly like to applaud the authors on the manuscript, which includes a series of rigorous experiments, is clearly written and presented, and presents interesting results that further our knowledge of mutational hotspots in bacteria. The primary result of this work is that a C->T mutational hotspot found at nucleotide position 565 in the gene nlpD is driven by transcription. The authors show that nlpD C565 is additionally situated within the promoter region of the neighbouring stress response gene rpoS. They report a positive correlation between induction of the rpoS promoter region and rates of C565T mutation. They then use these results to estimate an increase in mutation rate of ~5700-fold over the genomic average mutation rate, which is reduced by ~60-fold when the rpoS positive regulator psrA is removed from the genome.

It is surprising that the hotspot mutation is both caused by transcription and causes an increase in transcription. However I think the authors have provided a set of experiments that show that this phenotype is coincidental and that mutation does precede selection. Most reassuring of these results is the Q189W (TGG) mutation, because while this mutation reduces transcription initially, it should not reduce levels of transcription once a reversion to TAG occurs. This strain therefore disentangles mutation rates and phenotype by only affecting transcription prior to the mutational event. This is different from the psrA knockout which removes rpoS transcription both preceding to and following the C565T mutation. My only reservation is therefore not with the effect but with the reported magnitude, as described below:

_

We thank reviewer #1 for their encouraging and considered comments. Agreed - It is remarkable that the hotspot caused by transcription drives a mutation which further increases transcription. We have tried to limit details of the consequences of this process to avoid distracting the reader from the core messaging of the manuscript (that the promoter has a hotspot and not the consequences of the hotspot). We also agree that the Q189W (TGG) mutation provides firm evidence to the presence of the hotspot independent to the nuances of the mutational reporter we have constructed.

_

Major comment (requires clarification or amendment):

Is a fluctuation assay followed by FALCOR analysis an appropriate test for determining the mutation rate of a sequence that is dictated by rpoS induction? RpoS is primarily active/induced in the stationary phase (Schuster et al., 2003; supported by Figure S1), and FALCOR follows the basic assumptions of the Lea‐Coulson Model (Hall et al., 2009). Therefore the assay as employed may violate the following assumptions of Lea-Coulson outlined by Foster, 2006:
1. The cells are growing exponentially. (For this assumption to not be violated the assay must be terminated as soon as exponential phase ends. As the increased mutation rate is driven by the induction of rpoS transcription, and the OD does not change between 18 and 22h (Figure S1), this is not the case.)
2. The probability of mutation per cell-lifetime does not vary during the growth of the culture. (Again, this assumption is violated by mutation rate increasing as rpoS is naturally induced toward the end of the assay).

As the input parameters of FALCOR are the number of mutants (r) within a given cell count (N), if cell divisions continue to occur after the exponential phase but the total N remains unchanged, this may lead to an overestimate in mutation rate. Conversely if mutation rate only increases toward the end of the assay this may lead to an underestimate of mutation rate.

The authors derive a 5700-fold increase based on their fluctuation assay compared against the rate determined by Long et al., 2018. This previous manuscript used a mutation accumulation experiment and determined mutation rate using the number of mutations and the number of elapsed generations. It is therefore not directly comparable to this study for the reasons stated above. I therefore suggest that the reported fold change of 7500 should either be tested more rigorously or clarified.

Could the authors either explain why their 7500-fold report is robust to the violated assumptions outlined above, or add these caveats/limitations when they report the fold change in mutation rate in the manuscript.

_

We agree with reviewer #1; is good to consider of appropriateness of the fluctuation assays used, and further to challenge the comparison of a fluctuation assay with mutation rates derived from mutation accumulation.

We will first address the appropriateness of the fluctuation assay using stationary phase cultures. Firstly, there is not a hard and fast moment when stationary phase has occurred. We could aim to take samples for a fluctuation assay when the cells are in true exponential phase (probably around hour 14-15 of the assay), but then we may not always have mutants in the culture and we may not have full induction of *rpoS.* For these reasons we chose a later time point when cultures entered stationary but were still growing. These later samplings also allow for predictable cellular concentrations and less wasteful plating across several dilutions. Practicality and demonstrability aside, reviewer #1 reasonably suggests these mutation rates would be underestimated if stationary phase and expression from the *rpoS* promoter specifically induced mutations (which we now explicitly make the reader aware of **lines** **254-265**).

(Amusing aside: these listed assumptions are often compromised. Even in foster (2006) it is stated that in fluctuation assays “The cultures are allowed to grow, usually to saturation…”) on the page proceeding the list of assumptions.)

Prompted by these comments, we now present direct assessment the temporal appearance of the C565T mutation in growing cultures (now included in **supp Figure S4).** This time course presents a measure of mutant frequency over time (measurements of mutation rate over time would require impractical levels of replication requiring thousands of plates and dozens of rounds of sanger sequencing). This data suggests high C565T mutation rates are not unique to stationary phase (based on the comparison of measures at ~14 hours and after 18 hours, see **Fig. S4**) though there appears to be a transient decrease at some of the early time points (~16 and ~17.5 hrs). If our main fluctuation assays were sampled earlier, this might have resulted in lower measures of mutation rate (by ~7-fold). We have now been more explicit that these measures are estimates sensitive to violations of assumptions (**lines 254-265**), and comparisons with genomic averages from mutation accumulation provide many causes of uncertainty (**lines 283- 290**). We have now dropped mention of “5700x” from the text, and primarily written the mutation rate is orders of magnitude higher than expected (**lines** **289** and **360**). We only retained an approximate value of ~5,000 x in the abstract (lines 32) where short summary of this relative rate is far more succinct than descriptions of relative magnitudes.

_

Minor comment and typo:

Line 61: Change ‘deamidation’ to ‘deamination’.

_

Thanks! Changed in **line 70.**

_

Lines 124-125: “a subtle change in the sequence of codon 189 dramatically reduces (but does not abolish) the extreme parallelism previously observed.”
If it is dramatically reduced it can no longer be ‘extreme’. Consider rephrasing to something like: “a subtle change … dramatically reduces the molecular parallelism at the codon, but it continues to mutate at higher frequency than other positions (exact binomial test, p value).”

_

Agreed: we have simply replaced with “the extreme parallelism previously observed” with the more accurate “mutational parallelism across replicate microcosms” (see **line 130-131).**

_

**Reviewer #2**: This paper investigates the processes driving the high genetic parallelism observed in cell chain phenotype mutants that evolve in static microcosms of Pseudomonas. The authors suggest that this parallelism is driven by a C-to-T mutational hotspot at position C565 in the nlpD gene, which does not confer a selective advantage. They propose that the elevated C-to-T mutation rate at this site is associated with transcriptional activity of the rpoS gene, whose transcription start site is located within the nlpD gene. The experiments are well done and the writing is lucid. However, I have several concerns about the data and interpretation, noted below.

Major comments

1. I am not convinced that there is no role for selection in explaining the observed genetic parallelism. Fig 2 shows that 5 of the tested nlpD mutations are less fit than C565T and three others have similar fitness. So, none of the tested mutations are more beneficial than C565T. The latter also seems to have a selective advantage compared to the ancestor. Though no statistical test is provided for this comparison, the authors say that the fitness advantage is mild. A mean fitness advantage of ~10%, seen in Fig 2, is far from mild. It is also worth discussing why the results for this competition assay are different from the one shown for the Kan assay (Fig S2, where the ancestor and C565T mutant have similar fitness). Page 9, line 227: the fitness effect of C565T is reported as near-neutral, whereas in Fig S2 the legend says that its fitness is significantly lower. Looking at Fig S2 I am surprised that there is indeed a significantly lower fitness of the C565T mutation; given the error bars I would expect it to be neutral. Please double-check.
Overall, I think all the evidence for a selective advantage of the C565T mutation is a little bit brushed under the rug, and the presented narrative and statements are too strong and not clearly supported by the data. Ideally one would want to try and quantify how much of the parallelism is explained by selection vs. mutation, as done in some earlier studies (though I am not sure this is possible here). As an aside, for the mutants in Fig 2, did these still show the CC phenotype even after the Q189W mutation was reversed?

_

Many thanks to Reviewer #2 for these deep considerations of the manuscript. After reading the results from this perspective, we agree there could be more nuanced description of the role of selection in the evolution of the C565T mutation (we have now made several edits to make this role clearer – see following). We are quite sure that selection plays a strong role in the initial evolution of the C565T mutation from the ancestral type (see lines **81-83** which is referring to Fig.3 in Lind et al). We have now edited lines **87-93** to clarify the likely molecular basis of this selective advantage. The fitness assay data clearly shows an advantage of the C565T mutant over many others (edited lines **158 to 162**, which might be explained by the transcriptional effects caused by the C565T mutation (edited lines **197 to 202**). We also agree that the selective benefit of the C565T over the ancestral is not mild (it was only mild relative to the other measures in **Fig 2**) and have deleted “mild” (see **Fig 2 legend**).

More generally, Reviewer #2’s comments highlight the need for a clear motivation and interpretation of **Fig. 2** – the data is not intended to indicate that selection if not important for the evolution of the C565T mutation. Rather, **Fig. 2** is intended to demonstrate that selection does not readily explain the repeated observation of the C565T mutation compared to “**all possible nlpD truncation mutants”** (**line 154**). That the C565T mutant of observed orders of magnitude more frequently across experimental microcosms completely conflicts with the similar measurable fitness to, for example, the C49T (17*) mutation, and only makes sense in the light of C565T being generated by a mutational hotspot. We hope the additions and edits to lines **152 to 168** and **197-202**, better presents our interpretation.

Regarding the neutral cost of the mutation (**line 256 - 257)**. These fitness measures (**Fig S2**) were of the C565T mutation which arise in the mutational reporter we used, not in the complicating context of nlpD. For the importance of accuracy during of the mutation rate, it is important that these mutants are not fitter than the ancestral type so that the mutation is not selected during the fluctuation assay, leading to overestimates of mutation rate. Thanks to these comments (and from other reviewers), and seeing the potential for confusion of the different fitness assays presented, we have expanded and clarified the need for assays which define the dynamic of the mutant reporter constructs (**lines 254-265).** And to quickly address the aside – yes, these mutants in Figure 2 were still CC mutants with the reversion of the Q189W mutation (**line 497**).

_

2. The mutation rate reporter used here is a little problematic. This assay can only detect mutations that will increase rpoS expression (and therefore provide Kan resistance). As a result, any mutations in nlpD that may occur at a comparable rate to C565T, but that have no effect on rpoS expression, will not be detected. So, at the moment it is not clear whether this specific C to T mutation has a higher rate compared to neighbouring positions; and this information is important to attribute the parallelism solely to an elevated mutation rate at that specific site. A second issue is that the mutation rate for C565T from this selection-based mutation rate assay is compared to a genome-wide C to T mutation rate obtained from previous mutation accumulation experiments. Given the large differences in culture conditions and assay methods, I would take the estimated differences in mutation rates with a helping of salt. I don’t think these are lethal problems — I agree that the evidence supports a mutational hotspot in this region — but I think more nuanced discussion, with clarity on the limitations of the assays and results, is warranted.

_

Yes, the mutational reporter is only intended to measure the rate of the C565T mutation – only as a coincidence did we find mutations in the neighbouring nucleotide which would cause kan resistance. Left unknown is the mutation rate across nlpD at other nucleotide positions to directly compare the mutation rates at C565 to other positions (such as in the 5’ region of nlpD). We have now explicitly discussed this in the discussion (**see edited paragraph lines 371-386**). We have also further emphasised in the discussion that the reporter allows the mutation rate to be measured with and without the induction of transcription by PsrA, so can claim that the mutation rates are influenced by the induction (which we consider our main contribution).

We also agree with reviewer #2 (who shared the view of reviewer #1) that methodological differences between our fluctuation assays and mutation rates derived from mutation accumulation assays causes difficulties in direct comparisons. However, the units of mutation rate used in the long study are identical to ours, and we are unaware of a more “comparable” estimate of average C to T mutation in P fluorescens SBW25. As per the response to Reviewer #1, we think it is helpful to guide the reader and provide a handle for the magnitude of this mutation rate compared to an average rate. In response to these comments, we have clarified that this is a separate technique and detailed the source of uncertainty associated with these comparisons (see **lines 284-290**). We have removed the specific numerical comparison in the results and discussion to reflect the intention of this comparison (lines **289 and 360**).

_

3. The proposed causal chain of events for high parallelism is not as clear as it could be. Initially I thought that the authors are proposing that because rpoS has generally high transcription, the mutation rate for C565T increases, which increases rpoS transcription further, reinforcing the mutational hotspot. This was puzzling, because the last two steps have no explanatory value for the mutational hotspot idea, since the 565 mutation has already occurred and that’s the end result one is trying to explain. Then I realized that these two things are decoupled, so that from the evolutionary parallelism perspective it is just a coincidence that the C565T mutation also increases rpoS transcription.
If I am understanding this correctly, I suggest that the text make this very clear, so as not to confuse readers.
If I am wrong and the two pieces are in fact connected in the authors’ minds, then those connections should be more clearly laid out, with clear support for the feed-forward loop for the mutational hotspot and its relevance to explain the parallelism.
More generally, if possible it would be good to induce overexpression of rpoS and then test if the mutation rate at C565T increases, as predicted. Providing (e.g., published) data on and discussion of the baseline rpoS transcription level, comparisons with other genes, and changes under experimental conditions, would also be useful.

_

Thanks to Reviewer #2 for prompting this clarification. We think reviewer 2 is more accurate in stating “these two things are decoupled, so that from the evolutionary parallelism perspective it is just a coincidence that the C565T mutation also increases *rpoS* transcription.” There might be some selective advantage to the C565T mutation resulting from increased transcription (and idea now expanded in lines **161-162** and **200-202),** but we have not speculated further that the very high transcription of the C565T mutation results in higher mutation rates at this locus. We hope the clearer description of the interaction of selection and mutation rates clarifies this point. We could speculate that even higher transcription levels from *rpoSp* from the C565T mutation would result in higher mutation rates which would revert the mutation, but a) higher mutation rates may result in various other mutations (not necessarily to WT), and b) it’s not clear if we could make a mutational reporter that would determine the rates of reverting mutations from C565T to wildtype. In short, we think we’d best avoid speculation of the adaptive consequences of this mutation (as per our responses to reviewer #1).

Additionally, though we like the idea of inducing *rpoS* (and have previously searched for ways to do exactly this), it’s impractical with the limited understanding of PsrA’s interaction with the *rpoS* promoter. There is evidence of post-translational activation of PsrA which is outside of our experimental control (edited **lines 332-334**, and **398-405**). However, we did the inverse successfully and removed PsrA to the expected effect (mutation rates were reduced as per **Fig 4**).

_

4. I am a little worried about the variation in the fitness data, which are key to the conclusions. E.g., the very large error bars in the fitness experiments (Fig 2), which in some cases go all the way from s=-0.3 to -0.5 across replicates. Is there a known reason for this large variation across replicates? It appears very high to me, but I could be mistaken, so a comparison with previous studies would be useful. Similarly, the clear marker effects in some cases are a bit worrisome (e.g., Fig S2). Was the latter accounted for in the statistical analysis (i.e., was the marker effect included as a random factor, using a mixed model)?

_

Correct: there is considerable variation in these assays when you compare them to fitness assays in shaken environments. Fitness measures of ‘wrinkly spreader’ types in static microcosms (see Lind et al., *eLife*, 2015, DOI: 10.7554/eLife.07074) have similar levels of variation. These heterogeneous environments likely allow variation in the carrying capacity of niches, and there’s always a chance for stochastic events such as “mat collapse” (establishing biofilms fall down). This variation is true biological variation between replicate populations and is not caused by statistical uncertainties due to low counts of cells (here at least 20,000 cells were counted). **Fig S2** gives an idea how variation is with SBW25 strains in shaken environments (with variation reduces ~10 fold). And regarding **Fig S2**: yes, with the lack of variation among replicates allows potential marker effects, however, these effects are nullified by the swapping of each marker in the background of both competitors (with equal replicate number) and so such marker effects should not compromise our non-parametric test (we didn’t use linear models). And more fundamentally, in neither marked background is there a tendency for the C565T mutants to be fitter than the ancestral reporter (so the mutant is not being selected during fluctuation assays – the motivation for this assay was edited in **lines 254-265**).

Minor comments

1. Page 2, line 52: The statement “The mechanistic causes of mutational hotspots are often idiosyncratic and unpredictable” is not well-supported by the following sentences, which discuss common reasons for mutational hotspots. Consider rephrasing to align with the provided examples of not so common mechanisms.

_

Agreed. We have replaced “and unpredictable” has been replaced with “various and idiosyncratic” (**line 63**).

_

2. Page 3, line 89: The sentence “single codon in nlpD is prone to mutate at high frequency” may be misleading since not all nucleotides in that codon exhibit a high mutation rate. It would be more accurate to say “single nucleotide in nlpD...”

_

Agreed. Corrected, see **line 95**.

_

3. Page 5, line 125: The sentence “This shows that a subtle change in the sequence of codon 189 dramatically reduces (but does not abolish) the extreme parallelism previously observed” could suggest that the Q189W mutant may confer some fitness benefit. Could the higher frequency (~20%) of this mutant in the SBW25 ∆wss nlpD Q189W background be due to a slight fitness advantage?

_

It is possible that the ‘SBW25 ∆wss nlpD Q189W’ genotype has some difference in fitness in static microcosms relative to ‘SBW25 ∆wss’, which might affect the ability for adaptive nlpD mutants invade the population. It is for that precise reason that the different nlpD mutants evolved from this genotype were reconstructed into a background without the Q189W, preventing any epistatic interactions and allowing fair comparisons to be made between the mutation presented in figure 2 with the C565T mutant. This has been emphasised with textual changes in in **lines** **156 to 158**.

_

4. Page 12, line 313: Typo: Change “in” to “on”.

_

Thanks! Corrected **lines 368**.

_

5. Line 225: “in the reporter” should be “in nlpD”?

_

It should be in the reporter (not in the wild type context) **line 293**, but note this paragraph is now heavily edited.

_

6. This recent paper presents some parallels, and might be relevant to discuss: Rodriguez, A. et al. Synonymous codon substitutions modulate transcription and translation of a divergent upstream gene by modulating antisense RNA production. Proc. Natl. Acad. Sci. 121, e2405510121 (2024).

_

Thanks - Interesting paper! There’s quite a growing body of evidence to suggest that de-novo promoters are easier to come by than expected (as the authors demonstrate with some elegant genetics). We tried to find a relevant section in our discussion, however, the involvement of anti-sense RNA (rpoSp regulates mRNA, albeit with internal regulatory features) and the do-novo nature of the promoter (the rpoS promoter is highly refined and regulated) a makes it challenging to include - but we’ll remember this for future studies on promoter evolution.

_

Reviewer #3: This study by Farr et al. further investigates a frequent mutational site in the nlpD gene of Pseudomonas fluorescens that can be selected by growth in static cultures. This mutation, a C to T transition at nucleotide ?? leads to a “cell chaining” phenotype, due to lack of cell separation at division. Interestingly, this mutation coincides with a predict promoter region for the downstream rpoS gene, encoding the general stress response sigma factor. The authors rule out fitness explanations for the frequent mutation. The data presented here clearly establish that C565T is a bona fide mutational hotspot, whose “hotness” requires transcription. The mechanism of this mutational event will therefore be of general interest. In addition, since many gram negative bacteria carry the same nlpD rpoS arrangement, with a promoter for rpoS internal to nlpD, this could be a more general phenomenon.

_

We thank reviewer #3 for their consideration of this work. Indeed, one of the motivating factors of this work has been not only the remarkably predictable nature of this mutation, but that it occurs in such a conserved region – allowing future comparisons and measures of this phenomena in other model species.

_

specific comments;
line 24 the term “molecular parallelism” is probably not familiar to most readers, please define

_

To remove this jargon – and also prevent any confusion about the role of selection on the evolution of these mutants (raised by reviewer #2) – this sentence has been replaced with “Fitness measures of alternative *nlpD* mutants did not explain the deterministic evolution of C565T mutants” (**lines 23-24).**

_

line 129 Figure 1: Showing the nucleotide sequence as in Fig S3 would be helpful here.

_

We tried to place S3’s larger sequence context in the boxes in Fig 1, but it started to become cluttered and less clear what the mutation is altering (we aimed to show both nucleotide and amino acid changes). We have made a smaller textual change in the figure legend to make clear that references to CAG and TAG are “nucleotide” sequence specific. We also agree the reader would be helped by seeing the greater context of this mutation in a main figure. The promoter becomes a focus of study later in the text, and we have now included the sequence in figure 3C to make it clearer that this mutation’s position relative to predicted regions of the *rpoS* promoter (in particular the -10 region), which we now explore in more detail in the text (see paragraph **lines 204-215**)

_

Line 184 please cite stationary phase induction results here, as presented in Fig S1.

_

The assays in Fig S1 specifically match the exact conditions used in the fluctuation assays (inoculation of ~1000 CFU / mL), and indicate the appropriate sampling times of the fluctuation assay in order to have cells inducing rpoSp activity. Describing those assays later in the text allows the different methodology to be described and justified. Prompted by this comment (and in response to Reviewers #1’s comments) we clarified the justification for this time-course/induction preliminary experiment in lines **254 to 265**.

_

line 181 and Fig 3 please explain the relevance of the wss mutation, perhaps briefly in the intro while introducing the Lind et al 2017 results.

_

We have now edited the explanation of SBW25 𝛥*wss* to explain the function of *wss* after the first mention of the gene (see **lines 109-111**).

_

line 214 and later. The kan selection results section needs to be expanded. Did all kanR colonies have a mutation in the nlpD region? Results from the Sanger sequencing of kanR isolates should be presented somewhere and the fraction of these with C565T should be stated. A bit more experimental description of how the mutation rate was derived would be helpful in the results section (rather than just in the methods).

_

We are happy to share more details of the main results. Please see a new paragraph (**lines 267 to 278**) which details the conduct of the fluctuation assay (note the previous paragraph is also highly edited in response to reviewers’ comments). In this new paragraph we have described the number of mutant colonies confirmed to have the C565T mutation. We have also provided reference to both the raw data (which was previously available under “data availability”), and have a new table in supplementary table ‘**Table S3**’ (in submitted supplementary files) which depicts the CFUs and frequencies of mutants used to calculate mutation rates.

_

line 223 “Interestingly, these fluctuation assays revealed a mutational hotspot slightly broader than base-pair 565 of nlpD” How so? Please show data.

_

We found A564G mutants neighbouring the C565 nucleotide which presumably also activate rpoSp transcription : please see details in edited lines **276 to 277** and **290 to 295** and the new **Table S3** for the frequency of the mutants.

_

General comments about Discussion and Sankar results.
The authors cite the similarity between this hotspot and one observed in the thyP3 promoter of Bacillus subtilis. The Sankar et al. interpretation, which appears to be shared by the authors of this study, is that transcription promotes mutation, probably by promoting deamination (adenine for Sankar and cytosine here).

However, an alternative explanation (and to my mind, equally likely) is that the hotspot is resistant to correction by cellular surveillance pathways. Indeed the hotspot in the Sankar study goes away in mismatch repair deficient strains, a result consistent with the hypothesis that the hotspot change in not correctable (or corrected incorrectly to the mutated rather than parental base). Especially because there are transcription-coupled repair pathways (nucleotide excision repair, base excision repair and mismatch repair), this should be considered and stated as a possibility. A deaminated cytosine on the non-transcribed strand could be fixed to thymine(uracil) by removal of the parental guanine on the transcribed strand by transcription-coupled mismatch repair. This is why other mutations that arise in the promoter region (see comment above) would be interesting.

Doing the kan selection in a number of interesting mutants (mutL, mfd) would be important. Also , seeing whether C565T is stimulated by nitrous acid (a deamination reagent) and, if so, is it affected by psrA or mutL, may yield some mechanistic insight.

_

Thanks to Reviewer #3 for these helpful and perceptive comments!

First a general comments about this section of the discussion: we admit this section was too brief and didn’t convey sufficient nuance in suggesting underlying mechanisms of the C565T mutation. We mentioned the work of Sankar et al because of the similarities in the position and magnitude of the hotspot, and had only briefly mentioned another likely alternative “that the presence of PsrA may introduce a secondary effect and amplify mutation rates – such as blocking the mis-match repair process by MutS or MutL”. This may have conveyed the impression that we lean towards the explanation of Sankar et al for accounting for the mutagenesis of the C565T mutational hotspot. We do not lean towards either hypothesis, as this would require an uncertain period of experimental work – if they can be done at all.

Regarding the proposed assays: we also thank reviewer #3 for some of the suggested methods of disentangling these hypotheses (which we find an interesting idea). What is not clear to us is whether testing knockouts of mutational repair with combinatorial treatment of both solvents and induction by PsrA, will allow definitive conclusions about whether C565T is caused by inhibition of repair, solvent attack, or both. Those assays would only happen after systemic deletion of the MMR pathways and nucleotide lesion pathways before testing the effect of these deletions on mutation rate. There are also significant technical issues which result from deleting repair mechanisms: As per Sankar et. al., possible outcomes of deleting such repair pathways (such as MMR) is the production of mutations elsewhere in the reporter, making measurement of the hotspot impossible. We are also doubtful that transcriptional repair mechanisms would play a role in causing C565T, given the mutation is outside the transcript of rpoS. In summary, we agree that identification of the mechanism of this mutation process would be a significant contribution, but it would require substantial exploratory work not necessary for the conclusions of this manuscript, although it may form the basis of interesting future work.

We are very happy to correct the too brief treatment of the causes of the C565T mutation in the discussion. We have now described in greater detail the potential mutagenesis mechanism (see paragraph **388 to 410**) stressing these are two processes are possible or may even be co-occurring. The C565T hotspot is further distinguished from that in Sankar et al – *rpoSp* is positively induced by PsrA, providing an extra interaction with surveillance machinery than just the holoenzyme (Sankar and colleagues used a lac-based system).

Also, to address the specific point about what we can learn from other mutations detected by the mutational reporter – as per our comments to reviewer #2, the reporter may only select for transcription promoting mutations (nonsynonymous functional improvements to the *kanR* gene may be possible we just haven’t selected them). Regardless, the mutation we did find was the A564G mutation neighbouring the C565 base-pair (occurring at 1/10th the frequency). If deamination, for example, results in the A564G mutation, the frequency of this difference between deamination at 564 vs 565 might have something to do with the specific positioning of sigma factor binding (DOI: 10.1038/s41594-024-01349-9). However, we’d rather not speculate - the unusual sequence of the *rpoSp* (with high CG content) reduces certainly to the exact positioning of sigma factor binding and would require resolution with a different structural biology approach of which we have neither means nor experience.

_
