# Supplementary material for: An extreme mutational hotspot in nlpD depends on transcriptional induction of rpoS
Source: PLoS Genet. 2025 Jan 31;21(1):e1011572. doi: 10.1371/journal.pgen.1011572 (PMC11838912; doi:10.1371/journal.pgen.1011572)
Supplement: Attachment — Submitted filename: 8_1_25_Response to reviewers comments.docx [file pgen.1011572.s011.docx]

**8_1_25**

**Manuscript ID: PGENETICS-D-24-01022R1**

**Authors response to reviewers for** “**An extreme mutational hotspot in *nlpD* depends on transcriptional induction of *rpoS*”. Authors responses are between underscores in arial 11.**

_

**Comments to the Authors:**

**Reviewer #1**: Reviewer #1: I thank the authors for addressing the raised comments by myself and the other reviewers. The limitations of the experiments and the remaining questions are now more clearly presented. As for my major comment regarding the reported magnitude of mutation, I support the amendments and additions to the main text and the inclusion of Sup Fig 4. I do have two minor requests, which I believe will increase the clarity of the estimated rate without requiring a litany of additional text:

1. Line 29: "Fluctuation assays demonstrate the C565T mutation rate to be ~5,000-fold higher than expected". Change "demonstrate" to "estimate".

Your reply/sup fig 4 shows that terminating the assay at an earlier timepoint could lower estimated rates by ~7-fold. This is quite significant in the eyes of the reader, as ~5000-fold above average rates falls to ~800-fold. Therefore I think it's important to establish that this fold change is an estimate in the abstract.

_

No problem – “estimate” is now used.

_

2. Line 353: Could a comment be added that links psrA knockout rate to the expected rate, something like: "This represents a 60-fold decrease compared to the rate of mutation in SBW25 (~5000-fold to ~80-fold higher than expected T->C rates)."

The "60-fold reduction" of the psrA knockout is never mentioned in relation to the average rate. I feel the way the rates are currently reported makes them seem high when you want them to and low when you want them to. I.e. the hotspot is ~5000-fold higher than average (makes the rate seem super high) and then psrA knockout is ~60-fold lower than the hotspot (significantly lower, strongly implicating transcription as a causal factor). What is missing is any mention that the psrA knockout is still estimated to be ~80-fold higher than the average rate. Of course this can be readily calculated by the reader but mentioning it seems fair, because at the moment one could incorrectly assume that transcription alone can change mutation rates by ~5000-fold.

_

That’s a fair inclusion – we now state “This represents a ~60-fold decrease compared to the rate of mutation in SBW25; the ~5000-fold elevated rate of the C565T mutation is reduced to ~80-fold”.

_
